# Phenylalanine and Tyrosine as Feed Additives for Reducing Stress and Enhancing Welfare in Gilthead Seabream and Meagre

**DOI:** 10.3390/ani11010045

**Published:** 2020-12-29

**Authors:** Natalia Salamanca, Inmaculada Giráldez, Emilio Morales, Ignacio de La Rosa, Marcelino Herrera

**Affiliations:** 1 IFAPA Centro Agua del Pino, El Rompido-Punta Umbria rd., 21459 Cartaya, Spain; marcelino.herrera@juntadeandalucia.es; 2 Faculty of Experimental Sciences, University of Huelva, 21071 Huelva, Spain; giraldez@uhu.es (I.G.); albornoz@uhu.es (E.M.); 3 Escuela Superior de Ingeniería, University of Huelva, 21071 Huelva, Spain; ignacio.delarosa@dcaf.uhu.es

**Keywords:** fish, stress, welfare, phenylalanine, tyrosine, amino acid, feed additive

## Abstract

**Simple Summary:**

Food additives (phenylalanine and tyrosine) were tested in gilthead seabream (*Sparus aurata*) and meagre (*Argyrosomus regius*) to improve fish welfare in sea farms. These amino acids improved the stress response in both species, reducing some stress markers, though interspecific differences were detected. The results indicate that these dietary supplements could be provided before submitting fish to typical stress conditions in sea farms (sampling, grading, etc.) for improving animal welfare.

**Abstract:**

Increased aquaculture production is associated with a growing interest in improving fish welfare. For this reason, the search for strategies to mitigate stress has intensified, one of these strategies being food supplementation with amino acids. The objective of this study was to evaluate the effects of dietary phenylalanine (Phe) and Tyrosine (Tyr) on the stress response and metabolism of juvenile gilthead seabreams (*Sparus aurata*) and meagres (*Argyrosomus regius*). Fish batches were fed a control diet and two diets supplemented with 5% Phe or Tyr for seven days. At the end of the experiment fish were stressed by air exposure for 3 min and then sacrificed for the extraction of blood and brain. Classical plasma stress markers were analyzed (glucose, lactate, proteins, cortisol), as well as hormones derived from those amino acids (adrenaline, noradrenaline and dopamine). Despite interspecific differences, fish fed the diets supplemented with Phe or Tyr showed a reduction on several stress markers. However, interspecific differences were detected for many indicators. Concretely, hormonal stress markers were significantly attenuated in meagres fed the enriched diets. Moreover, the stress condition favored a mobilization of amino acids towards the brain, especially in supplemented diets, hence this amino acid excess could be used as an energy substrate to cope with stress.

## 1. Introduction

The gilthead seabream (*Sparus aurata,* Linnaeus 1758) and the meagre (*Argyrosomus regius,* Asso 1801) are aquaculture species with a high market value on the Mediterranean coast, and their cultivation is carried out by different commercial sea farms [1]. As the production of these species has increased, finding ways to minimize stress and increase animal welfare have become key issues in aquaculture studies.

The stress response in fish has been studied depending on numerous stressors such as salinity, transport, stocking density, temperature or air exposure [2,3,4,5,6]. Additionally, the attenuation of acute stress in fish using anesthetics, essential oils or feed additives has been extensively studied [7,8,9,10,11,12].

Protein is the most expensive part of fish diets and supplies amino acids (AA) for energy, growth, protein synthesis, and as substrates for key metabolic pathways. Functional AA is a term used to describe AA that are involved in cellular processes apart from protein synthesis. A deficiency or imbalance in functional AA may impair body metabolism and homeostasis. Recently, a growing interest in AA for enhancing disease resistance, immune response, reproduction, behavior and more has been demonstrated. This has led to a boost of commercially available functional fish feeds that aim to optimize fish performance and improve flesh quality [13]. For instance, it has been shown that diets enriched with tryptophan (Trp) could attenuate the stress response in meagre, although the involved metabolic pathways have not been described [14]. Furthermore, these diets can affect the immune response in this species [15].

Nevertheless, the effects of diets supplemented with other AA such as phenylalanine (Phe) and tyrosine (Tyr) have hardly been studied in any animal species. Phe is an essential amino acid so it can only be incorporated through diets. This amino acid is metabolized by two routes, oxidation to the Tyr and transamination to phenyl pyruvate, forming part of the classic pathways of metabolism [16]. Tyr is a direct precursor of catecholamine hormones and influences the formation of thyroid hormones (triiodothyronine and thyroxine). In this sense, it has been reported that plasma thyroid hormone concentration is useful for detecting stress in *Rutilus rutilus caspicus* [17]. In addition, Tyr can be catabolized to hydroxyphenyl pyruvate, becoming part of the energy metabolism. 

In mammals, a Phe/Tyr enriched diet mitigates the effects produced by an acute stressor [18,19,20]. Cotoia et al. [21] have shown that a metabolite of Phe, hydroxyphenyl pyruvate, improves survival at the cellular level under stress conditions in rats. However, there is no previous work on the metabolism of both amino acids in our objective species and there is no information on the catabolism, the physiological roles or the tissue accumulation of these substances. In fish, only two works have been published using Phe/Tyr supplements. In white seabream larvae (*Diplodus sargus*) both AAs decreased cases of bone deformity and mortality [22], and Herrera et al. [23] verified the mitigating effect of Phe on stressed cods (*Gadus morhua*).

Therefore, the objective of the present study is to understand the influence of a diet supplemented with Phe/Tyr on fish stress response and metabolism.

## 2. Materials and Methods 

### 2.1. Experimental Culture and Sampling

Gilthead seabreams (*Sparus aurata*), with an average weight of 65.00 ± 1.38 g (mean± standard error, SE) came from CULMASUR (Isla Cristina, Spain), and meagres (*Argyrosomus regius*), with an average weight of 138.67 ± 27.07 g came from IFAPA Agua del Pino (Cartaya, Spain). Fish were stocked at 20 fish/tank in six 250 and 500 L flat bottom squared tanks, respectively, at a stocking density of 5.55 Kg m^−3^. The culture water was recirculated, the mean temperature, salinity and dissolved oxygen levels being 21 ± 1 °C, 37 ± 1 g L^−1^ and above 5 ppm, respectively. Before the experiment started, fish were fed commercial fish feed (L2 Alterna^®^ Skretting, Burgos, Spain), at 2% biomass daily rations. The experimental treatments consisted of three feeding types (duplicated): control, Phe-enriched feed and Tyr-enriched feed. Fish feed was provided through 24 h-clock feeders, and the ration adjusted to 1–2% tank biomass daily, checking for the presence of remaining feed daily in order to be sure that the feed was supplied in excess. The tanks were cleaned and checked daily so probable dead fishes were removed. The feeding trials lasted seven days.

At the end of the experiment (7 days) the fish were sacrificed with 2-phenoxyethanol (1 mL L^−1^) for the extraction of blood and tissues. Previously, 10 fish from each feed treatment were submitted to an air exposure stress (3 min) and sampled 30 min later [24]; 10 fish were also sampled in the basal state. Blood was collected by puncturing the caudal peduncle with 1 mL heparinized syringes (25,000 units of ammonium heparin/3 mL of 0.6% NaCl saline, Sigma H6279). Plasma was separated from cells by centrifugation of whole blood (3 min; 10,000× *g*; 4 °C), and stored at −80 °C until the analyzes were performed. The brain was removed from each fish and stored at −80 °C until analyzed.

### 2.2. Experimental Food Making

Commercial fish feed (L2 Alterna^®^ Skretting, Burgos, Spain) was used as the control diet. L-phenylalanine and L-Tyrosine (dry powder) were purchased from ThermoFisher (Kandel, Germany). The commercial control diet was finely ground and mixed with amino acids and later water (400 mL Kg^−1^ dry feed). The amount of phenylalanine and tyrosine in each experimental diet was 5% (on dry feed) except in the control diet which did not have any amino acid. The mixture was thread pelleted into 2 mm diameter and 20–25 cm length strips. These were cut to get 2–3 mm size pellets. Finally, these food strips were dried at 60 °C for 24 h and were stored at 4 °C.

### 2.3. Plasma Analysis

Plasma glucose, proteins, lactate, triiodothyronine (T3), thyroxine (T4) and catecholamines (adrenaline, noradrenaline and dopamine) levels were measured using commercial kits from Química Analitica Aplicada S.A. (QCA Glucose Liquid Ref. 998,225, QCA Total Proteins Ref. 997,180, Tarragona, Spain), Spinreact (Lactate Ref. 1,001,330, Barcelona, Spain), Labor Diagnostika Nord (T3 ELISA 2nd Generation Ref TF E-2300, T4 ELISA 2nd Generation Ref TF E-2400), and 2-CAT (A-N) Research ELISA (Ref. BA E-5400, Nordhorn, Germany) adapted to 96-well microplates [24]. All assays were performed with a Tecan Sunrise microplate reader, using Magellan v2.5 software for Windows (Tecan Austria, Salzburg). Plasma cortisol levels were quantified by an ELISA kit (EA65, Oxford Biomedical Research, MI, USA) modified and adapted to fish [25]. Cortisol was extracted from 20 μL plasma in 200 μL diethyl ether. The lower limit of detection (88.2% of binding) was 0.005 ng mL^−1^ plasma. The inter-assay coefficient of variation was 9.8%, while the mean intra-assay coefficient of variation was 4.6%. The mean percentage of recovery was 90%. The main cross-reactivities (>5%; given by the supplier) were detected with prednisolone (66.9%), 11-deoxycortisol (58.1%), cortisone (15.9%), prednisone (13.7%), and 17-hydroxyprogesterone (5.4%).

### 2.4. Tissue Analysis

For brain catecholamines, 20–50 mg of brain tissue was homogenized by ultrasonic disruption with 150 µL of perchloric acid (PCA), and then mixed with 150 µL of potassium dichromate 0.15M. Following this, the mixture was centrifuged (1200× *g*; 4 °C; 10 min) and the supernatant removed. The precipitate was air-dried for 2 h at 25 °C. Finally, 250 µL of distilled water was added to the tube and the solution used for ELISA kit determination according to the above Section 2.3 Plasma analysis.

For analyzing Phe and Tyr metabolite concentrations in brain, tissues were dissolved in an acid hydrolysis and basic hydrolysis. The solution was diluted 1:20 in order to avoid high concentration. For derivatization, an aliquot (100 µL) of phenylalanine and tyrosine standard solution or sample was placed in a 2 mL vial, adding 400 µL of a water:ethanol:pyridine (60:32:8) mixture and 40 µL of ethyl chloroformate. It was capped and vigorously shaken using a vortex mixer for 30 s at room temperature. Then, 200 µL of chloroform (containing 1% ECF) was added, and the derivatives were extracted into the organic phase by striking the tube against a pad for about 30 s. The organic phase was dried with anhydrous sodium sulphate. The organic layer was transferred into a new vial with a 300 μL fixed insert.

Aliquots (1 μL) of the derivatized extracts were injected into a Shimadzu GC-MS TQ (GCMS-TQ8030) equipped with an Agilent HP-5MS fused silica capillary column (60 m × 0.25 mm i.d., 0.25 mm film thickness). The gas chromatograph system was equipped with a split/splitless injection port operating in splitless mode. The column was kept at 120 °C for 1 min, ramped at 5 °C min^−1^ to 200 °C, then the temperature was increased to 260 °C at 30 °C min^−1^ and held for 10 min, and finally ramped at 10 °C min^−1^ to 280 °C and held for 3 min. The carrier gas was helium with a constant flow of 1.2 mL min^−1^. The temperatures of the injector, transfer line and ion source were maintained at 250, 280 and 230 °C, respectively, and a solvent delay of 4 min was selected. The mass spectrometer was performed with electron ionization (EI) at 70 eV, operating in selected-ion monitoring (SIM) mode. The MS was tuned to m/z 69, 219 and 502 for EI corresponding to perfluorotributylamine (PFTBA). Each compound was identified using three characteristic ions, a quantifier and two qualifier ions, and the relative intensity of qualifier to quantifier ion (±20%). For phenylalanine m/z 176 (quantifier), m/z 102, m/z 91, and tyrosine m/z 107 (quantifier), m/z 192, m/z 264 were used. Quantification was conducted by the external standard method following the same procedure for all samples. The detection limits of phenylalanine and tyrosine were 0.35 and 0.05 ng mL^−1^, respectively [26,27].

### 2.5. Statistical Analysis

Normality and homoscedasticity of all data sets were checked through the Kolmogorov–Smirnov and Levene tests, respectively (SPSS v.21.0, IBM, Armonk, NY, USA). Differences among treatment were detected through a one-way ANOVA (normal variables) or Kruskal–Wallis (non-normal variables) tests, followed by Duncan or U–Mann–Whitney post-hoc tests. Data are expressed as mean ± standard error of mean (*n* = 7–10). The significance level was 0.05.

## 3. Results

### 3.1. Metabolite Analysis

**Figure 1:**
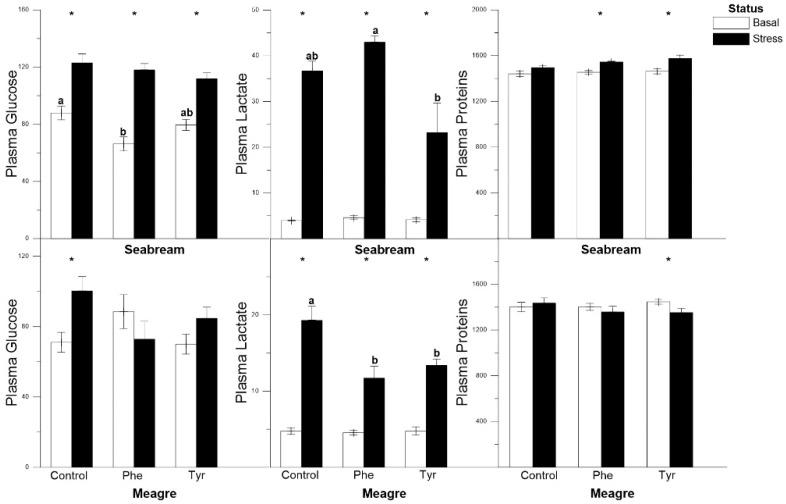
Plasma glucose, lactate and proteins concentrations (mg dL^−1^) for each treatment (mean ± SE, *n* = 10). The white and black bars are the basal and stress values, respectively. Asterisks indicate significant differences between the basal and stress conditions. Different letters indicate significant differences among feeding treatments within every condition.

In gilthead seabream, glucose and lactate showed significant differences between basal and stress levels for every type of diet, including the control. However, plasma protein concentration did not vary significantly for the control feed. In meagre the glucose levels were significantly higher only for those specimens subjected to stress and fed the control diet, and the lactate levels were significantly higher in stressed fish for every diet. Proteins showed significantly higher levels in the basal treatment for those specimens fed the Tyr diet. Overall, at basal or stressed status, there was no significant differences were registered between feed types for every metabolite (Figure 1).

### 3.2. Plasma Cortisol and Catecholamines

**Figure 2:**
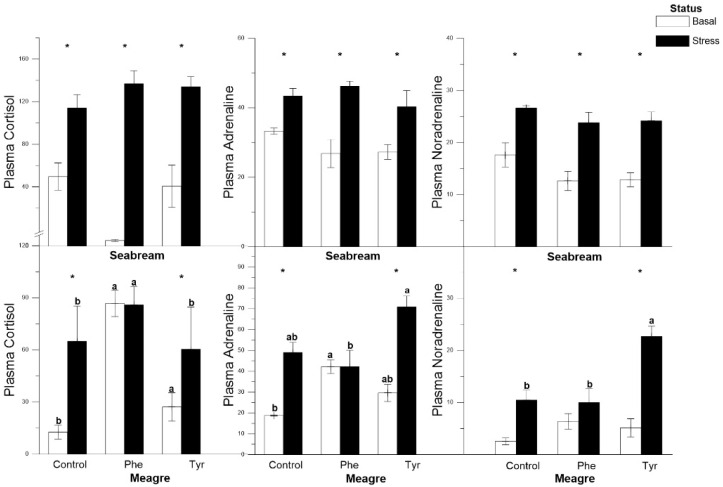
Plasma cortisol, adrenaline and noradrenaline concentrations (ng mL^−1^) for each treatment (mean ± SE, *n* = 10). The white and black bars are the basal and stress values, respectively. Asterisks indicate significant differences between the basal and stress conditions. Different letters indicate significant differences among feeding treatments within every condition.

In gilthead seabream, the variation among the three hormone levels followed a similar pattern whence the hormone levels were significantly higher under stress than the basal ones for every treatment. However, in meagre, the cortisol and catecholamine levels did not change significantly between basal and stress levels for the Phe treatment. In this species, the differences between the basal and stress states for noradrenaline were not significant for the Phe feed treatment (Figure 2).

### 3.3. Thyroid Hormones

**Figure 3:**
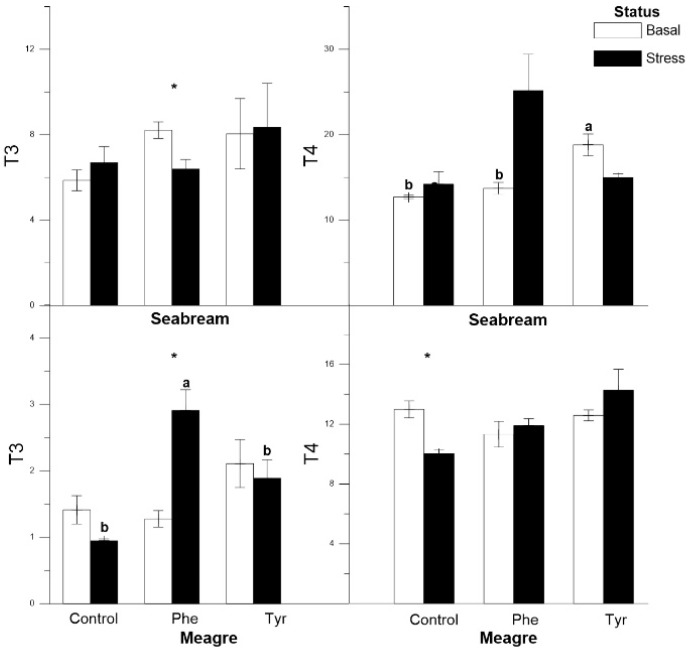
Plasma T3 and T4 concentrations (ng mL^−1^) for each treatment (mean ± SE, *n* = 10). The white and black bars are the basal and stress values, respectively. Asterisks indicate significant differences between the basal and stress conditions. Different letters indicate significant differences among feeding treatments within every condition.

The T3 levels in gilthead seabreams subjected to stress were lower than basal in the Phe diet (Figure 3). The levels of T4 remained significantly stable between stress and basal conditions, though T4 increased significantly in the Tyr treatment under basal status. In meagre fed the Phe diet, T3 values were significantly higher under stress. Both thyroid hormones did not vary significantly in stressed meagres fed the Tyr diet.

### 3.4. Tissue Analysis 

**Figure 4:**
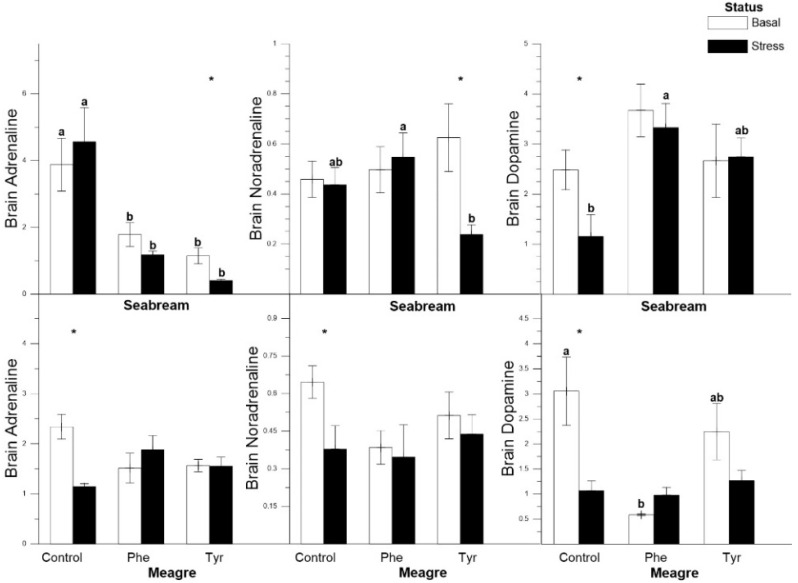
Brain adrenaline, noradrenaline and dopamine concentrations (ng mL^−1^), for each treatment (mean ± SE, *n* = 10). The white and black bars are the basal and stress values, respectively. Asterisks indicate significant differences between the basal and stress conditions. Different letters indicate significant differences among feeding treatments within every condition.

In gilthead seabream, adrenaline levels in stressed fish fed the Tyr and Phe diets were significantly lower than basal ones, and significant differences between basal and stress levels were only detected in the Tyr diet. Noradrenaline concentration showed the lowest value for the stressed fish in the Tyr treatment. Dopamine values were only different between stressed and basal gilthead seabreams in the control group. In meagre, catecholamine variations followed a similar pattern within every feed type, with significant differences only being registered in the control group, where basal concentrations were higher than stressed ones (Figure 4).

**Figure 5:**
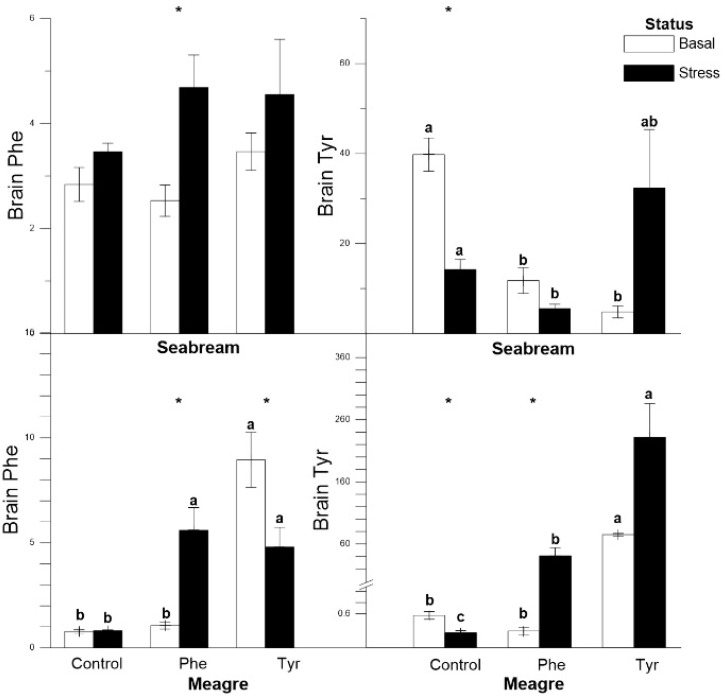
Brain Phe and Tyr concentrations (ng mg^−1^) for each treatment (mean ± SE, *n* = 10). The white and black bars are the basal and stress values, respectively. Asterisks indicate significant differences between the basal and stress conditions. Different letters indicate significant differences among feeding treatments within every condition.

In gilthead seabream, brain Phe concentration in fish fed the Phe diet increased significantly in the basal group, while it remained stable in the rest of the treatments. At the basal state, the Tyr concentration was significantly higher in the control group. In meagre, brain Phe and Tyr content increased significantly in stress status for the Phe diet, while they remained stable in the control group. Brain Tyr content in the Tyr diet was the highest in both the stress and basal states (Figure 5).

## 4. Discussion

In our study, the diets enriched with phenylalanine and tyrosine modulated the classic stress and hormonal markers. Furthermore, it seems that there was an interspecific variation since the supplemented diets did not affect both species in the same way. The accumulation of Phe and Tyr in the brain in some cases acted in a limiting way for the formation of catecholamine and thyroid hormones, which produced a decrease in them.

To date, the effects produced by the Tyr-enriched diet on the stress system have not been studied in any fish species; only the effect of this amino acid on the growth of *Diplodus sargus* larvae has been studied [22]. However, supplementation with Phe for mitigating stress has been evaluated in a few fish species [23,28,29]. Herrera et al. [23] concluded that the effects produced by Phe after acute stress caused a reduction in classical stress markers. In addition, Ren et al. [28] and Zehra and Khan [29] stated that an excess of Phe in a long-term diet produced nutritional stress and a decrease in growth parameters. However, those findings are not comparable to ours since the present experiment was a short-term experiment in which growth differences were not detectable, as it was only one-week in duration. In the present work, despite detecting inter-specific different patterns, both the Phe and Tyr additives modulated the stress response in meagre and gilthead seabream. However, meagres fed the Phe diet showed a more significant reduction or stabilization of plasma stress markers (hormones and metabolites). 

According to Herrera et al. [23] the values of the plasma stress markers were reduced when the diet was supplied with a high content of Phe in cods (*Gadus morhua*). A similar pattern was detected in our study, in which there was a reduction in the values of glucose and lactate, except for in the gilthead seabream. Cortisol in gilthead seabream specimens did not present a significant variation, however, in meagre specimens there was a decrease in cortisol values in fish fed the Phe diet compared to those fed the Tyr-enriched or control diet (Table 1). Therefore, it could indicate a stress attenuation due to amino acid supplements as described in previous works [14,19,28]. These effects on stress markers may be because of the fact that Phe and Tyr are precursors of key hormones and neurotransmitters that are involved in stress processes, such as adrenaline, noradrenaline, and dopamine [29].

Previous studies in gilthead seabream have indicated that air exposure for 3 min is effective in promoting stress system activation [30]. The stress response involves the recognition of a threat by the central nervous system, with a rapid increase in plasma catecholamines. In turn, the activation of these endocrine pathways derives from plasma and tissue metabolite changes to cope with the energy imposed by the stressor. In teleost fish, the activity of the hypothalamic–pituitary–interrenal (HPI) axis, involved in the stress response, is stimulated by noradrenaline [31,32]. Nevertheless, it has been reported that treatment with L-dopa (a precursor of dopamine) counteracts the elevation of plasma cortisol activity in *Salvelinus alpinus* [33,34]. Therefore, the relationship between HPI activity/cortisol release and catecholamine production in fish remains unclear.

In our stress conditions, plasma adrenaline and noradrenaline values in meagres fed the Phe-enriched diet were lower than those obtained for the Tyr-enriched diet (Figure 2). This may be because there is a relationship between the increase in the concentration of Phe and the decrease in the values of noradrenaline and dopamine [35]. Since by increasing the concentration of this amino acid in the blood, its passage through the blood–brain barrier is favored versus other amino acids, including tyrosine, and therefore catecholamine synthesis is not stimulated. However, this did not happen in the Tyr-enriched diet as it appears to have favored the catecholamine catabolism pathway. On the contrary, it seems that diets supplemented with Phe and Tyr in gilthead seabream slightly modulated plasma concentrations of adrenaline and noradrenaline. This could be due to the fact that Tyr did not stimulate the production of catecholamine hormones in favor of thyroid hormone synthesis. However, in the diet enriched with Tyr, the catecholamine catabolism pathway was favored, probably due to the fact that, in the absence of an increase in Phe concentration, this amino acid does not displace Tyr.

The level of dopamine present in the brain of the gilthead seabream was higher in both supplemented diets with respect to the control, thus amino acids enhanced this hormone response. According to Costa et al. [36], the effects of Phe supplements on the stress system are based in the dopamine production increase, though brain dopamine was not affected by the Phe enrichment in our experiment. However, the brain concentration of noradrenaline appeared to be slightly affected since it was lower in the specimens fed the Tyr diet than the other two diets. Gilthead seabreams fed the control diet presented the highest values with respect to the diets supplemented with amino acids in adrenaline values (Table 1). This may, as previously described, be due to the fact that diets supplemented with Phe could reduce the values of those hormones [33]. This was shown in our study since the highest catecholamine values occurred when brain Phe presented its minimum values. This does not seem to be the case with Tyr since this amino acid increase derived from higher levels of catecholamine hormones. In this sense, according to Li et al. [31], the amount of Tyr in the diet was limited the production of catecholamine hormones.

According to Peter [37], an increase in adrenaline produces an increase in plasma T4 levels. This occurred in our work for both species; in this sense it has been shown that adrenaline treatments activate the thyroid axis in climbing perch (*Anabas testudineus*) as evident in the rise of plasma T4 [38]. T3 levels were lower since this hormone is a precursor of adrenaline and, consequently, there was an increase in adrenaline values. In addition, there was a relationship between plasma cortisol levels and T3 since cortisol could stimulate the production of T3 [38]. Nevertheless, the effects of the experimental diets on these hormones were not clear for both species in the present work.

There was an increase in the Phe and Tyr brain concentrations under stress depending on which amino acid the specimens were fed, regardless of the species (Table 1). It seems that, overall, the stress conditions favored a mobilization of amino acids towards the brain, especially in supplemented diets. Again, the interspecific differences were evident, with the meagre supporting that statement more accurately. Since catecholamine hormones production from amino acids takes place in the interrenal tissue, this mobilization could be due to the use of Phe and Tyr as energy substrates to cope with stress, which is an energy-demanding process [16,21,23].

## 5. Conclusions

To our knowledge, this is the second work on the effects of Phe and Tyr additives on the stress response in fish. In spite of interspecific differences detected, both amino acids altered the stress response, mainly in meagre. Hormonal stress markers were significantly attenuated in meagres fed the enriched diets (Table 1). This fact could be the basis for further works on the study of these amino acids’ effects in situations of chronic stress, where their influence on growth and zootechnical variables as well as long-term physiological effects could be detected. 

## Figures and Tables

**Table 1:** Different types of stress response for each stress marker analyzed in the plasma and brain of gilthead seabream (*Sparus aurata*) and meagre (*Argyrosomus regius*) in the basal and stressed states. Fish were fed control diets (Ctrl) or diets supplemented with phenylalanine (Phe) or tyrosine (Tyr). C: no significant differences between baseline and stress states; R↑/R↓: increasing/decreasing response, significant changes between baseline and stress states.

Items	Gilthead Seabream			Meagre		
	Ctrl	Phe	Tyr	Ctrl	Phe	Tyr
Plasma glucose	R↑	R↑	R↑	R↑	C	C
Plasma lactate	R↑	R↑	R↑	C	C	C
Plasma proteins	C	C	C	C	C	C
Plasma cortisol	R↑	R↑	R↑	C	C	R↑
Plasma adrenaline	C	C	C	C	C	C
Plasma noradrenaline	C	C	C	C	C	C
Plasma T3	C	R↓	C	C	R↑	C
Plasma T4	C	C	C	R↓	C	C
Brain adrenaline	C	C	R↓	R↓	C	C
Brain noradrenaline	C	C	R↓	R↓	C	C
Brain dopamine	R↓	C	C	R↓	C	C
Brain Phe	C	C	C	C	C	C
Brain Tyr	C	C	C	C	C	C

## Data Availability

Data available on request due to restrictions.
